# Healthy dietary patterns decrease the risk of colorectal cancer in the Mecca Region, Saudi Arabia: a case-control study

**DOI:** 10.1186/s12889-017-4520-4

**Published:** 2017-06-29

**Authors:** Firas S. Azzeh, Eyad M. Alshammari, Awfa Y. Alazzeh, Abdelelah S. Jazar, Ibrahim R. Dabbour, Hani A. El-Taani, Ahmed A. Obeidat, Fayrooz A. Kattan, Sufyan H. Tashtoush

**Affiliations:** 1 Department of Clinical Nutrition, Faculty of Applied Medical Sciences, Umm Al-Qura University, P.O. Box: 7067, Makkah, 21955 Saudi Arabia; 2 Department of Clinical Nutrition, Faculty of Applied Medical Sciences, University of Ha’il, Ha’il, Saudi Arabia; 3 Department of Nutrition and Food Technology, Faculty of Agriculture, Mutah University, Alkarak, Jordan; 4 Department of Medical Oncology, Oncology Center, KAMC-HC, Makkah, Saudi Arabia; 5 Department of Clinical Nutrition, Faculty of Applied Medical Sciences, Taibah University, Yanbu, Saudi Arabia; 6 Clinical Nutrition Department, KAMC-HC, Makkah, Saudi Arabia

**Keywords:** Black tea, Coffee, Colorectal cancer, Dairy food, Leafy vegetables, Legumes, Olive oil

## Abstract

**Background:**

Colorectal cancer (CRC) is the first most common cancer in males and the third most common cancer in females in Saudi Arabia. Dietary habits are strongly associated with the inhibition or proliferation of malignancy. Therefore, this study is aiming to investigate the risks and protective benefits of dietary factors affecting CRC in the Mecca region of Saudi Arabia.

**Methods:**

A case-control study was conducted from June 2014 to March 2015. One hundred thirty-seven patients with colon and/or rectal cancer were recruited in the case group, while 164 healthy participants were recruited in the control group. A questionnaire was completed with the help of trained dietitians to study the effects of several dietary patterns on the risk of CRC.

**Results:**

Dairy product intake of 1–5 servings/day, legume intake of 3–5 servings/week, leafy vegetables intake of 1–5 servings/week, olive oil intake of 1–5 servings/week, black tea intake of three or more cups/day, and coffee intake of one or more cups/day was found to decrease the risk of CRC in participants.

**Conclusion:**

This study highlights the importance of changing dietary habits to decrease CRC incidence in the Mecca region.

## Background

Colorectal cancer (CRC) is one of the leading causes of death. It was responsible for approximately 70,000 deaths worldwide in 2012, according to World Health Organization (WHO) [1]. Globally, CRC is the third most common cancer in men and the second most common in women [2]. In Saudi Arabia, CRC has been reported to be the most common cancer in males and the third most common cancer in females, regardless of the age group, as it was diagnosed in 10.4% of all new Saudi cancer cases [3].

Following a healthy dietary pattern has been shown to decrease the incidence of CRC [4]. Therefore, determining the risk factors related to CRC is a crucial matter. Several studies have evaluated the influence of lifestyle factors, particularly nutrition on the development and prevention of CRC [5–8]. The probable risk factors associated with CRC are high fat diet, red and processed meat intake, overweight and obesity, diabetes, smoking, alcohol consumption, and physical inactivity [9–12]. Possible preventative foods for CRC include a high intake of fish, polyunsaturated fatty acids, milk, dietary fiber, whole grains, fruits, non-starchy vegetables, garlic, and cruciferous vegetables [7, 13, 14]. In Saudi Arabia, Alamri et al. concluded that inadequate intake of dietary antioxidants was considered as one of the main risk factors for CRC in Saudis, while the use of nonsteroidal anti-inflammatory drugs exert a protective effect [8].

According to the variation in traditions, beliefs, cultures, lifestyle, and dietary habits that affect food choices and behaviors among countries, the risk factors of CRC may differ among countries and among regions in the same country. However, no detailed studies were found in the Mecca region to determine the risk factors associated with CRC, even though, Mecca represents the fifth city in Saudi Arabia in CRC incidence [3]. Therefore, the aim of this study was to investigate the possible CRC dietary risk or protective factors in the Mecca region.

## Methods

### Subjects

A case-control study was carried out from June 2014 to March 2015 in the Mecca region of Saudi Arabia. One hundred thirty-seven patients with colon and/or rectal cancer (51.1% males, 56 ± 13.4 years of age) were recruited from King Abdullah Medical City Hospital (KAMC), Mecca, Saudi Arabia. Case subjects were compared with 164 control participants (49.4% males, 56.7 ± 8.7 years of age) from the same region. Both groups were matched for their age and gender. The inclusion criteria for the case group were Saudi nationality and CRC diagnosed in any region of the colon and/or rectum, whereas the exclusion criteria were people younger than 40 years of age and older than 75 years of age as well as patients diagnosed with other types of cancer. Controls were recruited from patients’ visitors and hospital staff.

### Data collection

Eligible participants were asked to complete a questionnaire under the supervision of trained registered dietitians that included personal information as well as information regarding nutritional habits. Usual food intakes and nutritional habits were assessed using an existing validated dietary questionnaires, as described in the Nashar and Almurshed investigation [15], including questions about daily intake frequency of fruits and vegetables, dairy products, poultry, and red and processed meat, as well as black tea (*Camellia sinensis*) and coffee. Black tea and coffee are usually served in Saudi traditional standard cups of 80 ml and 50 ml, respectively. Intake of fish and seafood, legumes, green leafy vegetables, and olive oil were measured on a weekly basis. Type of bread usually eaten per day (white or brown) was also evaluated. White bread is the bread made from white flour, while the brown bread is the bread made only from whole-wheat flour. Participants were educated about the serving size of each food item before beginning the questionnaire. Weight and height were measured by the dietitians using Detecto physician’s scale available at the hospital (Detecto, Webb City, Missouri, USA). Body Mass Index (BMI) was calculated as weight in kg divided by height in meters squared.

### Statistical analysis

Statistical analyses were performed with SPSS software (Statistic Package for Social Sciences) version 20. A t-test was used to explore the differences between case and control groups for parametric variables, and comparison of the categorical variables was performed by the chi-squared (*x* ^2^) test. The odds ratio (OR), 95% confidence interval (CI), and beta coefficient (β) were measured by logistic regression to determine the potential independent risk factors correlated to CRC. All variables were adjusted for the potential confounders as recognized in Tayyem et al. [4] study, which are age (continuous), gender, BMI (continuous), education, income, employment, smoking, marital status, physical activity, and family history of CRC (see Table 1). A *P*-value less than 0.05 was considered statistically significant. The Cochran-Armitage test was conducted using SAS (Statistical Analysis System; version 9.3) to determine whether there is a linear trend at *P* < 0.05 of diet effect on decreasing the incidence of CRC.

**Table 1:** Baseline characteristics of participant in the study group (*n* = 301)

Parameter	Control	Case
Age (year)	56.7 ± 8.4	56 ± 13.4
(BMI (kg/m^2^)	28.3 ± 5.5	25.9 ± 6.9
Employment (%)		
Yes	45.9	19.6
No	8.6	25.9
Education (%)		
Illiterate	0.3	16.3
Primary	1.0	9.3
Intermediate/secondary	7.3	8.3
Postsecondary	45.8	11.7
Family income in SR (%)		
<5000	4.7	16.3
5000–10,000	17.3	9.3
10,000–20,000	23.3	8.3
>20,000	9.3	11.5
Married (%)		
Yes	51.8	41.9
No	2.6	3.7
Family history of CRC (%)		
Yes	9	7.3
No	45.5	38.2
Smoking (%)		
Yes	7.9	6.7
No	46.5	38.9
Regularly exercise (%)		
Yes	22.6	13
No	31.9	32.5

Percentages are determined from the total (*n* = 301)

*Abbreviations: BMI* Body Mass Index, *SR* Saudi Riyals

## Results

The mean BMI of participants was 28.3 ± 5.5 kg/m^2^ and 25.9 ± 6.9 kg/m^2^ for control and case subjects, respectively. Table 2 shows the dietary habits of participants for the main food groups. Results showed that consumption of dairy products (servings/day) and legumes (servings/week) were different (*P* < 0.05) between participants in the control and the CRC groups. However, no differences (*P* > 0.05) were detected in consumption of red and processed meat (servings/day), poultry (servings/day) as well as fruits and vegetables (servings/ day) between the two groups. In addition, there were no differences (*P* > 0.05) with respect to type of bread usually eaten (white or brown) between the study groups (Table 2).

**Table 2:** Dietary habits for the main food groups between case and control participants

Parameter	Control (*n* = 164)	Case (*n* = 137)	*P*-value
Fruits and vegetables (servings/day)			
< 1	22 (13.4%)	14 (10.2%)	0.589
1–2	107 (65.3%)	97 (70.8%)	
3–5	34 (20.7%)	24 (17.5%)	
> 5	1 (0.6%)	2 (1.5%)	
Dairy products (servings/day)			
< 1	3 (1.8%)	13 (9.5%)	<0.001
1–2	130 (79.3%)	117 (85.4%)	
3–5	28 (17.1%)	6 (4.4%)	
> 5	3 (1.8%)	1 (0.7%)	
Red and processed meat (servings/day)			
< 1	11 (6.7%)	12 (8.7%)	0.091
1–2	98 (60%)	76 (55.5%)	
3–5	40 (24.2%)	26 (19%)	
> 5	15 (9.1%)	23 (16.8%)	
Poultry (servings/day)			
< 1	114 (69.5%)	96 (70%)	0.341
1–2	17 (10.4%)	19 (13.9%)	
3–5	26 (15.8%)	19 (13.9%)	
> 5	7 (4.3%)	3 (2.2%)	
Legumes (servings/week)			
< 1	8 (4.9%)	20 (14.6%)	0.006
1–2	103 (62.8%)	88 (64.2%)	
3–5	44 (26.8%)	22 (16.1%)	
> 5	9 (5.5%)	7 (5.1%)	
Bread type			
White	66 (40.2%)	59 (43.1%)	0.353
Brown	98 (59.8%)	78 (56.9%)	

Values are expressed as frequency (%)

*P*-values are obtained by *x* ^2^ test

Dietary habits of common foods and drinks of the study groups are shown in Table 3. Results showed that consumption of leafy vegetables (servings/week), olive oil (servings/week), black tea (*Camellia sinensis*; cup/day) and coffee (cup/day) were different (*P* < 0.01) between participants in the two groups. However, no differences (*P* > 0.05) were detected with respect to the fish and seafood consumption. Participants in the control group were more likely to consume leafy vegetables than the CRC group. The percentage of participants in the control group who consumed <1 serving per week of leafy vegetables was 4.9%; in contrast, 13.8% of participants in the CRC group consumed <1 serving per week of leafy vegetables. Participants in the control group were prone to consume more olive oil than those in the CRC group. Only 10.4% of participants in the control group consumed <1 serving per week of olive oil, while 30.6% of participants in the CRC group consumed <1 serving per week of olive oil. In addition, participants who consumed 1–2 servings/week of olive oil were 51.8 and 30.7% for the control and CRC groups, respectively. Furthermore, the percentage of subjects who consumed between 1 and 5 servings/week of olive oil was 70.3 and 47.5% for the control and CRC groups, respectively.

**Table 3:** Dietary habits for common foods and drinks of the study groups

Parameter	Control (*n* = 164)	Case (*n* = 137)	*P*-value
Fish and seafood (servings/week)			
< 1	20 (12.2%)	16 (11.7%)	0.37
1–2	34 (20.7%)	34 (24.8%)	
3–5	15 (9.2%)	24 (17.5%)	
> 5	95 (57.9%)	63 (46%)	
Leafy vegetables (servings/week)			
< 1	8 (4.9%)	9 (13.8%)	0.001
1–2	90 (54.9%)	74 (54%)	
3–5	46 (28%)	22 (16.1%)	
> 5	20 (12.2%)	22 (16.1%)	
Olive oil (servings/week)			
< 1	17 (10.4%)	42 (30.6%)	<0.001
1–2	85 (51.8%)	42 (30.7%)	
3–5	32 (19.5%)	23 (16.8%)	
> 5	30 (18.3%)	30 (21.9%)	
Black tea (cup/day)			
< 1	9 (5.5%)	25 (18.3%)	0.002
1–2	18 (11%)	21 (15.3%)	
3–5	21 (12.8%)	10 (7.3%)	
> 5	116 (70.7%)	81 (59.1%)	
Coffee (cup/day)			
< 1	5 (3%)	35 (25.5%)	<0.001
1–2	55 (33.5%)	25 (18.3%)	
3–5	16 (9.8%)	16 (11.7%)	
> 5	88 (53.7%)	61 (44.5%)	

Values are expressed as frequency (%)

*P*-values are obtained by *x* ^2^ test

Our results showed that healthy participants in the control group tended to consume more black tea (cup/day) than people suffering from CRC. The percentage of subjects in the control group who consumed <1 cup per day of black tea were 5.5, while 18.3% of subjects in the CRC group consumed <1 cup per day of black tea. In addition, results of this study showed that subjects in the control group tended to consume more coffee (cup/day) than those in the CRC group. Only 3% of the healthy subjects in the control group consumed <1 cup per day of coffee; however, the percentage of subjects from the CRC group who consumed <1 cup per day of coffee was 25.5%. In addition, 33.5% of subjects in the control group consumed 1–2 cups/day of coffee, while only 18.3% in the CRC group consumed 1–2 cups/day of coffee.

The potential independent variables correlated with CRC are shown in Table 4. The protective diet associations with decrease CRC incidence were found in the regular intake of 1–2 servings (OR = 0.2, 95% CI = 0.05–0.79, *P* = 0.022) and 3–5 servings of dairy products per day (OR = 0.06, 95% CI = 0.01–0.28, *P* < 0.001). In addition, weekly intake of 3–5 servings of legumes (OR = 0.28, 95% CI = 0.09–0.85, *P* = 0.025), 1–2 servings (OR = 0.28, 95% CI = 0.08–0.96, *P* = 0.04) and 3–5 servings of leafy vegetables (OR = 0.18, 95% CI = 0.05–0.67, *P* = 0.01), 1–2 servings (OR = 0.24, 95% CI = 0.11–0.52, *P* < 0.001) and 3–5 servings of olive oil (OR = 0.24, 95% CI = 0.1–0.61, *P* = 0.003) were associated with decreased risk of CRC. Results showed that daily intake of 3–5 cups of black tea (OR = 0.17, 95% CI = 0.05–0.56, *P* = 0.003) and more than 5 cups of black tea (OR = 0.21, 95% CI = 0.08–0.53, *P* = 0.001) were associated with lower CRC incidence. In addition, coffee intake of 1–2 cups (OR = 0.08, 95% CI = 0.03–0.25, *P* < 0.001), 3–5 cups (OR = 0.25, 95% CI = 0.72–0.95, *P* = 0.033) and more than 5 cups of coffee per day (OR = 0.11, 95% CI = 0.04–0.31, *P* < 0.001) were associated with decreasing the incidence of CRC. Our results also showed that there was a linear trend (*P*-trend <0.01) of lower CRC incidence with the increase in consumption of dairy products, legumes, black tea, and coffee.

**Table 4:** Potential dietary habits as predictors for CRC

Independent variable	β	OR	95% CI	*P*	*P*-trend
Dairy products (servings/day)					
< 1	0	1			<0.001
1–2	−1.60	0.2	0.05–0.79	0.022	
3–5	−2.91	0.06	0.01–0.28	<0.001	
> 5	ND	ND	ND	ND	
Legumes (servings/week)					
< 1	0	1			0.004
1–2	−0.75	0.48	0.17–1.31	0.149	
3–5	−1.28	0.28	0.09–0.85	0.025	
> 5	−0.33	0.72	0.16–3.32	0.673	
Leafy vegetables (servings/week)					
< 1	0	1			NS
1–2	−1.26	0.28	0.08–0.96	0.043	
3–5	−1.7	0.18	0.05–0.67	0.01	
> 5	−0.66	0.52	0.13–1.99	0.338	
Olive oil (servings/week)					
< 1	0	1			NS
1–2	−1.44	0.24	0.11–0.52	<0.001	
3–5	−1.41	0.24	0.1–0.61	0.003	
> 5	−0.57	0.57	0.23–1.37	0.205	
Black tea (cups/day)					
< 1	0	1			<0.001
1–2	−1.06	0.35	0.11–1.08	0.067	
3–5	−1.77	0.17	0.05–0.56	0.003	
> 5	−1.56	0.21	0.08–0.53	0.001	
Coffee (cups/day)					
< 1	0	1			0.002
1–2	−2.49	0.08	0.03–0.25	<0.001	
3–5	−1.37	0.25	0.72–0.90	0.033	
> 5	−2.23	0.11	0.04–0.31	<0.001	

The reference is the control group

*Abbreviations: β* beta coefficient, *CI* confidence interval, *ND* not determined, *NS* not significant, *OR* odds ratio

## Discussion

The age-standardized incidence rate of CRC in Saudi Arabia increased over the period between 1994 and 2010 by approximately two-folds, and had an estimated growth rate of 3.2% [3]. A report by the Saudi Cancer Registry showed that CRC was the most common cancer among males (11.8%) and the second most common cancer among Saudis of all ages [16]. Therefore, it is of critical importance to evaluate the risk and protective factors related to CRC in Saudi Arabia.

Study results showed that the main protective factor in decreasing CRC incidence was consuming healthy foods, primarily dairy products, legumes, leafy vegetables, olive oil, black tea, and coffee. Although it might seem that sun exposure in Saudi Arabia is high; however, due to cultural practices, unfortunately sun exposure among Saudis, especially women, is negligible. Most dairy products, especially liquid milk in Saudi Arabia is fortified with vitamin D, which signifies the potential importance of consuming vitamin D from dairy products in decreasing the incidence of CRC. Calcium and vitamin D contents in milk were shown to be inversely associated with CRC [7, 17, 18] by a variety of mechanisms that include enhancing apoptosis and cell differentiation. Calcium attaches the bile acids to free fatty acids, which would diminish their proliferative effect in colon mucosa [7]. High calcium and vitamin D consumption might result in reducing epithelial exposure to the toxic effect of free fatty acids by facilitating their binding with bile salts in the colon [18, 19]. In addition, increasing immune responses and the antioxidant effect of conjugated linoleic acid in dairy products were also related to a reduction in CRC risk [20], which might explain our results regarding the consumption of dairy products decreasing the incidence of CRC. From their recent meta-analysis, Song et al. [7] concluded that there was a probable negative association between milk and risk of CRC, and estimated relative risk (RR) to be 0.91 (95% CI = 0.85–0.94, *P* < 0.05). Another meta-analysis suggested that an intake of dairy products of 400 g per day is required to reduce CRC risk. However, since the total dairy intake in that study was below 200 g per day, this did not lead to a decrease in CRC incidents [13]. However, other studies, such as Bergsma-Kadij et al. [21] and Martinez et al. [22], found no conclusive evidence on the effects of calcium and vitamin D consumption on decreasing the incidence of CRC. The discrepancies in results were probably because the aforementioned studies had low intake of calcium and vitamin D in contrast to our study, which included a wide range of dairy food consumption. This was also demonstrated in the linear effect of increasing dairy products, which would indicate high dairy consumption and, hence, high levels of calcium and vitamin D consumption exert more protective effects on decreasing CRC incidence.

On the basis of our study results, consumption of coffee and black tea were negatively associated with CRC risk. Several antioxidant compounds and cancer chemopreventive constituents are well known in black tea such as catechins, mainly epigallocatechin gallate, which exerted strong antioxidant properties and CRC chemoprevention effects in both in vivo [23] and in vitro [24] studies. In addition, several studies such as those by Koňariková et al. [25] and Patel et al. [26] investigated the anticancer effect of black tea extracts against human carcinoma cell lines. Koňariková et al. [25] showed that black tea natural extracts were most effective in non-proliferation activity against human colon carcinoma cell HT-29 after 24 h of exposure. Apoptotic cell death in HT-29 cells were observed after 72 h incubation of repetitive exposure to black tea. In addition, the DNA of HT-29 carcinoma cells exhibited strand breaks and oxidative damage to DNA after 24 h incubation with black tea extracts. Furthermore, another study [27] showed that other black tea extracts such as theaflavin-2 induced apoptosis of human colon cancer cells. Patel et al. [26] showed that black tea extracts decreased tumor volume and multiplicity by inhibiting 1,2-dimethylhydrazine-induced colorectal tumorigenesis. However, in vivo cohort and cross-sectional studies on the effects of drinking black tea on the incidence of CRC have been inconsistent. Tea consumption was found to be associated with increased incidence of colon cancer in a cohort study of Finnish men [28]. A case-control study found that drinking two cups of tea or more decreased the incidence of CRC in Swedish participants [29]. Another study found that prevention of CRC was apparent when male participants consumed more than 1.5 cups of black tea per day [30]. Other studies found no association between the consumption of tea and CRC [31–34]. The inconsistencies found among the different studies might be attributed to the differences in geographical locations as well as time of consumption, since consumption of tea in the Middle East is habitual with or right after meals, which might have direct effect from black tea phenols. In addition, tea consumption in the Middle East is distinguishably higher than that of other communities, with more than 70 and 59% of participants in the control and CRC groups, respectively, consuming >5 cups of tea per day. However, other studies such as Michels et al. [33], Tavani et al. [34], and Terry et al. [28] did not categorize black tea consumption of more than 5 cups per day; instead, they pooled all participants who drank more than 2 cups of tea per day in one group. This might have concealed any significant effects of drinking more than 5 cups of tea per day in the previous results. This is also demonstrated with the significant (*P* < 0.001) linear trend of increasing tea consumption that leads to a decrease in CRC incidence. These habits may cause major differences among studies.

Although the mechanisms by which coffee may exert a protective effect against cancers of the lower gastrointestinal tract are not well defined, it has been suggested to be due to the antioxidant, antimutagenic, and anticarcinogenic effects of phenolic compounds in coffee. Coffee contains several phenolic compounds such as caffeic, chlorogenic, and cumaric acid, as well as melanoidins and diterpenes that include cafestol and kahweol compounds. These compounds are known to possess anticancer properties of many types of cancer including CRC [35, 36], while decaffeinated coffee was inversely correlated with rectal cancer [33], colon cancer [37], or CRC in European females [38]. A cohort meta-analysis found no association between coffee consumption and CRC in Finland [39]. Possible reasons for this discrepancy could be due to the method of coffee preparation, which is different among different cultures. When Li et al. [38] performed meta-analysis on subgroups based on geographic areas; they observed that there was a significant inverse correlation between coffee consumption and risk of CRC in Europe, but not in America or Asia. They suggested that this was because most communities in Europe consume unfiltered boiled ground coffee, a practice which is not common in America and Southeastern Asia. The filtration of boiled ground coffee might render the anti-carcinogenic properties in coffee ineffective due to the removal of diterpene compounds by filtration [39]. However, in Finland, more than 80% of the population consumed filtered coffee; which might explain the ineffectiveness of coffee in decreasing CRC incidence in the Finnish sample. In the Middle East, people consume boiled unfiltered coffee, which would keep diterpene compounds in coffee liquid and thus might lead to decreasing the incidence of CRC. This might clarify the discrepancy between our results and those that found ineffectiveness of coffee in decreasing CRC incidence.

As a part of Mediterranean diet, legumes, leafy green vegetables, and olive oil, could be negatively associated with CRC [40]. These food groups contain powerful antioxidant, anticancer, anti-inflammatory, and antiproliferative properties [40–42]. The most important bioactive constituents with anti-CRC possibility are genistein and isoflavones in soy beans, quercetin and isothiocyanates in broccoli, apigenin in parsley and celery, oleuropein in olive oil, and ellagic acid and hydroxycinnamic acids (p-coumaric, caffeic, schlorogenic, m-coumaric, and ferulic acids) in most leafy vegetables [19, 41–44]. A cohort study [45] showed that consumption of more than two serving of legumes per week was associated with a decreased risk of CRC (RR = 0.53, 95% CI = 0.33–0.86) relative to persons who consumed legumes less than once a week. Another meta-analysis of cohort studies reported that a decreased risk of CRC (RR = 0.85, 95% CI = 0.72–1.00) was associated with legume fiber intake [43]. This protective effect of legume fibers could be attributed to several factors, such as the presence of flavonoids and isoflavonoids [46], which would inhibit the growth of malignant cells and induce cell differentiation, increase carcinogen excretion by increasing stool bulk, and delay transit time by diluting the carcinogenous compounds in the stool or by binding bile acids [47–50]. Furthermore, legume fibers may stimulate the anaerobic fermentation of gut microflora, resulting in the formation of short-chain fatty acids, which induce apoptosis in CRC cells [51]. Additionally, legumes are rich in several antioxidants such as vitamin E and selenium and vitamin B_6_, as well as lignans that might contribute to the reduction of CRC risk [52]. The significant (*P*-trend = 0.006) linear trend of increasing legume consumption (servings per week) possibly indicated a cumulative effect of anticarcinogenic compounds and legume fibers. A recent in vitro study [53] showed that certain legumes such as lupine seeds, chickpeas, soybeans, and fava beans were effective in inhibiting MMP-9 gelatinase protein, which has been found to be involved in the progression of CRC in both human and animal models [54, 55]. In addition, Lima et al. [53] found that legumes were effective in inhibiting HT-29 CRC cell line migration and growth.

Our results are in agreement with evidence in the literature that suggests a protective role of olive oil against CRC. A meta-analysis of 25 epidemiological studies showed that consumption of olive oil is promising in decreasing CRC [56]. An in vitro study showed that hydroxytyrosol, which is a phenolic compound present in olive oil, downregulated epidermal growth factor receptor (EGFR) expression in colon tumor cells [57]. Another in vitro study [58] found that olive oil resulted in initiation of apoptosis and cell differentiation in the HT-29 CRC cell line. In addition, COX-2, a cyclooxygenase isoform that is known to play a major role in colorectal carcinogenesis, was inhibited by an olive oil supplement. In an epidemiological study by Stoneham and his colleagues [59], olive oil consumption was negatively associated with the incidence of CRC.

In a meta-analysis of epidemiological studies, consumption of leafy vegetables was found to decrease the incidence of CRC [60], while a cohort prospective study [61] found that consumption of leafy vegetables and high-fiber grains decreased the risk of fatal colon cancer (*P*-trend = 0.031 in men and 0.0012 in women). Another cohort study found that consumption of brassica and leafy vegetables decreased the incidence of CRC. However, that previous study showed no association between consuming total vegetables or fruits on the incidence of CRC; which is in agreement with the results of our study.

There is currently increasing concern worldwide regarding the potential carcinogenicity in the colon and/or rectum of red and processed meat consumption [62]. From their meta-analysis, Song et al. [7] concluded that red and processed meat had a convincing positive level-of-evidence associated with CRC. A recent case-control study (3745 CRC cases and 6804 hospital controls) in Italy illustrated that the OR between low vs high consumption of meat was 0.86 (95% CI = 0.79–0.94). Study results did not provide a similar association for red meat and CRC [63]. Consumption of red meat in Saudi society is considered a common habit and tradition. However, the high daily intake of red and processed meat in CRC (> 1 serving/day = 91.3%) and control (> 1 serving/day = 93.3%) subjects did not support its effect on CRC incidence, a matter requires further investigation with a larger sample size.

This study is limited by determining the risk and protective factors of CRC for different food subgroups. Further studies are recommended to find the possible determinants of CRC with different meats, dairy products, legumes, fruits, vegetables, and other traditionally consumed food items in the Mecca region. In addition, the sample size was relatively small.

## Conclusions

In conclusion, study results demonstrated that the consumption of dairy products, coffee, black tea, legumes, leafy vegetables, and olive oil were shown to have protective effects against CRC in the Mecca region. However, there was no effect of the bread type, as well as consumption of red and processed meats, poultry, fish and seafood, and total vegetables and fruits on the incidence of CRC.

## Abbreviations

BMI: Body mass index
CI: Confidence interval
CRC: Colorectal cancer
KAMC: King Abdullah Medical City Hospital
OR: Odds ratio
SAS: Statistical analysis system
SPSS: Statistic package for social sciences
WHO: World Health Organization
*x*^2^: Chi-squared

## References

[1] World Health Organization. 2016. Cancer: Fact sheet N° 297 Updated February 2015. WHO. http://www.who.int/mediacentre/factsheets/fs297/en/. Accessed 28 Apr 2016.

[2] Al-Ahwal MS, Shafik YH, Al-Ahwal HM (2013). First national survival data for colorectal cancer among Saudis between 1994 and 2004: what’s next?. BMC Public Health.

[3] Alsanea N, Abduljabbar AS, Alhomoud S, Ashari LH, Hibbert D, Bazarbashi S (2015). Colorectal cancer in Saudi Arabia: incidence, survival, demographics and implications for national policies. Ann Saudi Med.

[4] Tayyem RF, Bawadi HA, Shehadah I, et al. Dietary patterns and colorectal cancer. Clin Nutr. 2017. In Press. 10.1016/j.clnu.2016.04.029 27206698

[5] Tayyem RF, Bawadi HA, Shehadah IN (2015). Macro- and micronutrients consumption and the risk for colorectal cancer among Jordanians. Nutrients..

[6] Murphy N, Norat T, Ferrari P (2012). Dietary fibre intake and risks of cancers of the colon and rectum in the European prospective investigation into cancer and nutrition (EPIC). PLoS One.

[7] Song M, Garrett WS, Chan AT (2015). Nutrients, foods, and colorectal cancer prevention. Gastroenterology.

[8] Alamri FA, Saeedi MY, Kassim KA (2014). Dietary and other risk factors for colo-rectal cancer in Saudi Arabia. J Med Med Sci.

[9] Deng L, Gui Z, Zhao L, Wang J, Shen L (2012). Diabetes mellitus and the incidence of colorectal cancer: an updated systematic review and meta-analysis. Dig Dis Sci.

[10] Aleksandrova K, Pischon T, Jenab M (2014). Combined impact of healthy lifestyle factors on colorectal cancer: a large European cohort study. BMC Med.

[11] Cho S, Shin A, Park SK, Shin H-R, Chang S-H, Yoo K-Y. Abstract 1734: Body mass index, physical activity and risk of colorectal cancer in the Korean Multi-center Cancer Cohort (KMCC). Cancer Res. 2016;76(14 Supplement). http://cancerres.aacrjournals.org/content/76/14_Supplement/1734. Accessed 1 May 2016.

[12] Tayyem RF, Bawadi HA, Shehadah I, et al. Meats, milk and fat consumption in colorectal cancer. J Hum Nutr Diet. 2016:1–11. 10.1111/jhn.12391 27302247

[13] Aune D, Lau R, Chan DSM (2012). Dairy products and colorectal cancer risk: a systematic review and meta-analysis of cohort studies. Ann Oncol.

[14] Maskarinec G, Jacobs S, Harmon BE (2016). Four a priori-defined diet quality indexes and survival among men and women with colorectal cancer: The multiethnic cohort. FASEB J.

[15] Nashar RM, Almurshed KS (2008). Colorectal cancer: a case control study of dietary factors, king faisal specialist hospital and researh center, riyadh, saudi arabia. J Family Community Med.

[16] Mosli MH, Al-Ahwal MS (2012). Colorectal cancer in the Kingdom of Saudi Arabia: need for screening. Asian Pacific J Cancer Prev.

[17] Davoodi H, Esmaeili S, Mortazavian AM (2013). Effects of milk and milk products consumption on cancer: a review. Compr Rev Food Sci Food Saf.

[18] Huncharek M, Muscat J, Kupelnick B (2009). Colorectal cancer risk and dietary intake of calcium, vitamin D, and dairy products: a meta-analysis of 26,335 cases from 60 observational studies. Nutr Cancer.

[19] Pericleous M, Mandair D, Caplin ME (2013). Diet and supplements and their impact on colorectal cancer. J Gastrointest Oncol.

[20] Bassaganya-Riera J, Hontecillas R, Horne WT (2012). Conjugated linoleic acid modulates immune responses in patients with mild to moderately active Crohn’s disease. Clin Nutr.

[21] Bergsma-Kadijk JA, van Veer P, Kampman E, Burema J (1996). Calcium does not protect against colorectal neoplasia. Epidemiology.

[22] Martinez ME, Willett WC (1998). Calcium, vitamin D, and colorectal cancer: a review of the epidemiologic evidence. Cancer Epidemiol Biomark Prev.

[23] Larsen CA, Bisson WH, Dashwood RH (2009). Tea catechins inhibit hepatocyte growth factor receptor (MET kinase) activity in human colon cancer cells: kinetic and molecular docking studies. J Med Chem.

[24] Du G-J, Zhang Z, Wen X-D (2012). Epigallocatechin gallate (EGCG) is the most effective cancer chemopreventive polyphenol in green tea. Nutrients.

[25] Koňariková K, Ježovičová M, Keresteš J, Gbelcová H, Ďuračková Z, Žitňanová I (2015). Anticancer effect of black tea extract in human cancer cell lines. Spring.

[26] Patel PK, Kommajosyula N, Rosebrock A (2008). The Hsk1(Cdc7) replication kinase regulates origin efficiency. Mol Biol Cell.

[27] Ar G, En Jao DL, Huang M-T (2011). Effects of the black tea polyphenol theaflavin-2 on apoptotic and inflammatory pathways in vitro and in vivo. Mol Nutr Food Res.

[28] Hartman TJ, Tangrea JA, Pietinen P (1998). Tea and coffee consumption and risk of colon and rectal cancer in middle-aged Finnish men. Nutr Cancer.

[29] Baron JA. Gerhardsson de Verdier M, Ekbom A. Coffee, tea, tobacco, and cancer of the large bowel. Cancer Epidemiol Biomarkers Prev. 1994;3:565–70. 7827586

[30] Su LJ, Arab L (2002). Tea consumption and the reduced risk of colon cancer - results from a national prospective cohort study. Public Health Nutr.

[31] Woolcott CG, King WD, Marrett LD (2002). Coffee and tea consumption and cancers of the bladder, colon and rectum. Eur J Cancer Prev.

[32] Terry P, Wolk A (2001). Tea consumption and the risk of colorectal cancer in Sweden. Nutr Cancer.

[33] Michels KB, Willett WC, Fuchs CS, Giovannucci E (2005). Coffee, tea, and caffeine consumption and incidence of colon and rectal cancer. J Natl Cancer Inst.

[34] Tavani A, Pregnolato A, La Vecchia C, Negri E, Talamini R, Franceschi S (1997). Coffee and tea intake and risk of cancers of the colon and rectum: a study of 3,530 cases and 7,057 controls. Int J Cancer.

[35] Cavin C, Holzhaeuser D, Scharf G, Constable A, Huber WW, Schilter B (2002). Cafestol and kahweol, two coffee specific diterpenes with anticarcinogenic activity. Food Chem Toxicol.

[36] Green CJ, de Dauwe P, Boyle T, Tabatabaei SM, Fritschi L, Heyworth JS (2014). Tea, coffee, and milk consumption and colorectal cancer risk. J Epidemiol.

[37] Sinha R, Cross AJ, Daniel CR (2012). Caffeinated and decaffeinated coffee and tea intakes and risk of colorectal cancer in a large prospective study. Am J Clin Nutr.

[38] Li G, Ma D, Zhang Y (2013). Coffee consumption and risk of colorectal cancer: a meta-analysis of observational studies. Public Health Nutr.

[39] Bidel S, Hu G, Jousilahti P (2010). Coffee consumption and risk of colorectal cancer. Eur J Clin Nutr.

[40] Bamia C, Lagiou P, Buckland G (2013). Mediterranean diet and colorectal cancer risk: Results from a European cohort. Eur J Epidemiol.

[41] Khanam U, Oba S, Yanase E, Murakami Y (2012). Phenolic acids, flavonoids and total antioxidant capacity of selected leafy vegetables. J Funct Foods.

[42] Deng G, Lin X, Xu X, Gao L, Xie J, Li H (2013). Antioxidant capacities and total phenolic contents of 56 vegetables. J Funct Foods.

[43] Zhu B, Sun Y, Qi L, Zhong R, Miao X (2015). Dietary legume consumption reduces risk of colorectal cancer: evidence from a meta-analysis of cohort studies. Sci Rep.

[44] Barbaro B, Toietta G, Maggio R (2014). Effects of the olive-derived polyphenol oleuropein on human health. Int J Mol Sci.

[45] Michels KB, Edward GE, Joshipura KJ (2000). Prospective study of fruit and vegetable consumption and incidence of colon and rectal cancers. J Natl Cancer Inst.

[46] Constantinou A, Kiguchi K, Huberman E (1990). Induction of differentiation and DNA strand breakage in human HL-60 and K-562 leukemia cells by genistein. Cancer Res.

[47] Stephen AM, Cummings JH. Mechanism of action of dietary fibre in the human colon. Nature 1980;284:283–4. 10.1038/284283a0 7360261

[48] Papas MA, Giovannucci E, Platz EA (2004). Fiber from fruit and colorectal neoplasia. Cancer Epidemiol Biomark Prev.

[49] Fleming SE, O’Donnell AU, Perman JA (1985). Influence of frequent and long-term bean consumption on colonic function and fermentation. Am J Clin Nutr.

[50] Bingham SA, Day NE, Luben R (2003). Dietary fibre in food and protection against colorectal cancer in the European Prospective Investigation into Cancer and Nutrition (EPIC): an observational study. Lancet.

[51] Emenaker NJ, Calaf GM, Cox D, Basson MD, Qureshi N (2001). Short-chain fatty acids inhibit invasive human colon cancer by modulating uPA, TIMP-1, TIMP-2, mutant p53, Bcl-2, Bax, p21 and PCNA protein expression in an in vitro cell culture model. J Nutr.

[52] Jang Y, Lee JH, Kim OY, Park HY, Lee SY (2001). Consumption of whole grain and legume powder reduces insulin demand, lipid peroxidation, and plasma homocysteine concentrations in patients with coronary artery disease: randomized controlled clinical trial. Arterioscler Thromb Vasc Biol.

[53] Lima AIG, Mota J, Monteiro SAVS, Ferreira RMSB (2016). Legume seeds and colorectal cancer revisited: Protease inhibitors reduce MMP-9 activity and colon cancer cell migration. Food Chem.

[54] Mook ORF, Frederiks WM, Van Noorden CJF (2004). The role of gelatinases in colorectal cancer progression and metastasis. Biochim Biophys Acta - Rev Cancer.

[55] Herszényi L, Hritz I, Lakatos G, Varga M, Tulassay Z (2012). The behavior of matrix metalloproteinases and their inhibitors in colorectal cancer. Int J Mol Sci.

[56] Pelucchi C, Bosetti C, Negri E, Lipworth L, La Vecchia C (2011). Olive oil and cancer risk: an update of epidemiological findings through 2010. Curr Pharm Des.

[57] Terzuoli E, Giachetti A, Ziche M, Donnini S (2016). Hydroxytyrosol, a product from olive oil, reduces colon cancer growth by enhancing epidermal growth factor receptor degradation. Mol Nutr Food Res.

[58] Llor X, Pons E, Roca A (2003). The effects of fish oil, olive oil, oleic acid and linoleic acid on colorectal neoplastic processes. Clin Nutr.

[59] Stoneham M, Goldacre M, Seagroatt V, Gill L (2000). Olive oil, diet and colorectal cancer: an ecological study and a hypothesis. J Epidemiol Community Health.

[60] Trock B, Lanza E, Greenwald P (1990). Dietary fiber, vegetables, and colon cancer: critical review and meta-analyses of the epidemiologic evidence. J Natl Cancer Inst.

[61] Thun MJ, Calle EE, Namboodiri MM (1992). Risk factors for fatal colon cancer in a large prospective study. J Natl Cancer Inst.

[62] World Health Organization. 2015. Links between processed meat and colorectal cancer, WHO statement. Updated 29 October 2015. http://www.who.int/mediacentre/news/statements/2015/processed-meat-cancer/en/. Accessed 1 May 2016.

[63] Filomeno M, Bosetti C, Bidoli E, et al. Mediterranean diet and risk of endometrial cancer: a pooled analysis of three Italian case-control studies. Br J Cancer. 2016:1–4. 10.1038/bjc.2015.153 PMC4647248 26010500

